# Abdominal Obesity is Associated with Peripheral Artery Disease in Hemodialysis Patients

**DOI:** 10.1371/journal.pone.0067555

**Published:** 2013-06-28

**Authors:** Peir-Haur Hung, Hung-Bin Tsai, Chien-Hung Lin, Kuan-Yu Hung

**Affiliations:** 1 Department of Internal Medicine, Ditmanson Medical Foundation Chia-yi Christian Hospital, Chia-yi, Taiwan; 2 Department of Applied Life Science and Health, Chia-Nan University of Pharmacy and Science, Tainan, Taiwan; 3 Department of Tramatology, National Taiwan University Hospital, Taipei, Taiwan; 4 Institute of Clinical Medicine, National Yang-Ming University, Taipei, Taiwan; 5 Department of Pediatrics, Zhongxing Branch, Taipei City Hospital, Taipei, Taiwan; 6 Department of Internal Medicine, National Taiwan University Hospital, Taipei, Taiwan; Pennington Biomedical Research Center, United States of America

## Abstract

**Background:**

Peripheral arterial disease (PAD) is a leading cause of morbidity in hemodialysis (HD) patients. Recent evidence suggests that abdominal obesity (AO) may play a role in PAD. However, the association between AO and PAD has not been thoroughly studied in HD patients.

**Methods:**

The present cross-sectional study aimed to examine the relationship between AO and PAD in a cohort of 204 chronic HD patients. The ankle brachial index (ABI) was used as an estimate of the presence of PAD. Plasma adiponectin levels, interleukin-6 (IL-6) levels, high sensitivity C-reactive protein (hs-CRP) levels, asymmetric dimethylarginine (ADMA) levels, and lipid profiles were measured. Logistic regression was used to estimate the association between the presence of PAD and AO as well as other potential risk factors.

**Results:**

The metabolic risk factors and all individual traits, including elevated ln-transformed hs-CRP, were found to be significant (*P*<0.05) more frequently in HD patients with AO than that in control subjects. Patients with AO had a higher prevalence of PAD than the control individuals, with a mean ABI of 0.96±0.23 and 1.08±0.16 (*P*<0.0001) and PAD prevalence of 26.9% and 10.8% (*P = *0.003), respectively. By multivariate analysis, AO (odds ratio [OR], 4.532; 95% CI, 1.765–11.639; *P = *0.002), elevated serum ln-transformed ADMA (OR, 5.535; 95% CI, 1.323–23.155; *P = *0.019), and ln-transformed IL-6 (OR, 1.567; 95% CI, 1.033–2.378; *P = *0.035) were independent predictors of the presence of PAD.

**Conclusions:**

HD patients with AO exhibited a cluster of metabolic risk factors and lower ABI. AO, elevated serum ln-transformed ADMA, and ln-transformed IL-6 were independent predictors of the presence of PAD.

## Introduction

Abdominal obesity (AO), known as an over-accumulation of visceral adiposity that can be estimated by waist circumference (WC), is prevalent in hemodialysis (HD) patients [1]. In the general population, AO is characterized by chronic low grade inflammation with increased serum inflammatory cytokine levels; it is considered to be a risk factor for atherosclerosis, cardiovascular disease, and increased mortality [2]. Postorino *et al.* have shown that high WC is associated with a high risk of cardiovascular mortality in patients with chronic kidney disease (CKD) as well as dialysis patients [3]. In addition, Witasp *et al.* [4] recently revealed increased proinflammatory gene expression in subcutaneous abdominal fat in patients with advanced CKD, providing a biological insight at the cellular level and linking obesity with inflammation in CKD patients.

Recent available data suggest that peripheral artery disease (PAD) is prevalent in HD patients and is a strong predictor for subsequent cardiovascular and overall mortality [5]–[6]. Although traditional cardiovascular disease risk factors such as smoking and diabetes are strong risk factors for PAD [7], only AO, but not generalized obesity, has been shown to be associated with PAD in the general population [8]–[9]. The association of AO and PAD in HD patients has not been elucidated thus far. The ankle brachial index (ABI) was reported to be a good marker for atherosclerosis and to be useful in the diagnosis of PAD [10]. Because prior findings demonstrated an association of AO with PAD in the general population, we hypothesized that HD patients with AO will have a higher prevalence of low ABI values and clinical PAD. In addition, we investigated the relationship between AO and various biochemical markers, which reflected the status of systemic inflammation and insulin resistance.

## Methods

### Study Population

This was a cross-sectional study conducted in the HD unit of a regional hospital in Taiwan. We recruited 204 patients who had received chronic HD treatment, 3 times a week for more than 3 months, with each session lasting for 4 h. Exclusion criteria included irregular or inadequate HD therapy with a mean Kt/V <1.2 within 3 months before entry, inability to measure WC and ABI, and evidence of hypercatabolic disease. The WC cutoff points were based on those for the Chinese population [11]. This clinical study followed the Declaration of Helsinki and was approved by the Ethics Committee.

### Laboratory Measurement

Biochemical and hematological parameters were obtained from midweek pre-dialysis blood samples. Venous blood samples were collected in the morning after an overnight fast. Plasma samples were separated from blood cells and stored at −70°C. For analysis, samples were centrifuged at 1500×*g* at 4°C for 10 min. Kt/V was calculated using Daugirdas’ second formula [12].

Levels of serum high-sensitivity C-reactive protein (hs-CRP) and insulin were measured by chemiluminescent immunoassays (Immulite 2000; DPC, Los Angeles, CA). Hemoglobin levels were measured by Sysmex XT-1800i (Sysmex America Inc., Mundelein, IL). Insulin sensitivity was quantified using the Homeostasis Model Assessment of Insulin Resistance (HOMA-IR) equation to measure fasting insulin and glucose levels (HOMA-IR = I×3 G/22.5), where I is insulin (lU/mL) and G is glucose (mmol/L) (IR: HOMA-index≥2.5 µU/mL×mmol/L) [13]. Fasting blood sugar, albumin, glutamic pyruvic transaminase (GPT), cholesterol, and triglyceride levels were measured with an automated analyzer (Hitachi 7170, Tokyo, Japan). For hs-CRP, the intra-assay coefficient of variance was 8.7%, sensitivity was 0.1 mg/L, and upper limit of detection was 150 mg/L [14]. Expected values for healthy individuals were hs-CRP≤3 mg/L [15]. Anti-HCV antibodies were measured using a third-generation enzyme immunoassay (Abbott Laboratories, North Chicago, IL). Serum pro-inflammatory cytokine levels were measured with high-sensitivity interleukin (IL)-6, tumor necrosis factor (TNF)-α, and adiponectin immunoassay kits. These measurements were based on a solid-phase sandwich enzyme-linked immunoassay with recombinant human IL-6 (normal range: 0.03–200 pg/mL; RayBiotech, Atlanta, GA), TNF-α (normal range: 0.48–100 pg/mL; RayBiotech), and adiponectin (normal range: 0.48–100 pg/mL; RayBiotech).

### ABI Measurement

The ABI index was measured in all participants and control individuals using a vascular screening device (VP 1000; Colin Corp. Co., Ltd, Komaki, Japan) that simultaneously measures the bilateral arm and ankle (brachial and posterior tibial arteries, respectively) blood pressure by an oscillometric method. The measurement was obtained after completion of the dialysis treatment and after allowing patients to rest in a supine position for at least 5 min. Some patients required more than 10 min for their blood pressure to stabilize. ABI was calculated by the ratio of the ankle systolic pressure and arm systolic pressure. The systolic pressure of the arm without dialysis access and the lower value of the ankle pressure were used for the calculation. Each patient’s ABI index was determined at least twice during different dialysis sessions, and the mean of the measurements was used for analysis. A criterion for the diagnosis of PAD was an ABI of <0.9 that may indicate varying degrees of atherosclerosis in the lower extremity arteries. Patients with an ABI of ≥1.3 were excluded, because this indicates poorly compressible leg arteries and inability to gauge arterial obstruction accurately [6].

### Statistical Analysis

Statistical analyses were performed with SPSS/Windows software (SPSS Science, v. 15.0, Chicago, IL). Each concentration of pro-inflammatory cytokines was ln-transformed to improve its level of normality. Data were analyzed by the *t*-test or χ^2^ test, depending on the nature of the variables. A Pearson’s correlation analysis was also performed to evaluate the relationship between the WC and various clinical factors. Consecutive logistic regression models (multivariate-adjusted) were constructed to confirm the independent association of AO and PAD.

## Results

The mean age of the 204 participants was 63.4±13.0 years, and 52.0% were women. All the patients had been on maintenance HD for a duration of 4.5±3.9 years. The mean WC was 90.6±7.3 cm in the group with AO (n = 93, 45.6%) and 77.6±7.4 cm in the group without AO (n = 111, 54.4%).

Comparisons of the demographic and laboratory data for the patients with and without symptoms of AO are shown in Table 1. There were no statistically significant differences in age, smoking habits, blood pressure, and diabetes. However, patients with AO were more likely to be female (58/93 vs 48/111, *P* = 0.006); further, they had a higher body mass index (BMI) (25.0±3.0 vs 20.6±3.1 kg/m^2^, *P*<0.001).

**Table 1:** Differences of clinical and biochemical parameters in hemodialysis patients between abdominal obesity and non-abdominal obesity.

	Patients with abdominal obesity (n = 93)	Patients not with abdominal obesity (n = 111)	*P* value
Age (years)	64.8±11.7	62.3±14.0	0.164
Male (%)	35 (37.6%)	63 (56.8%)	0.006
HD years	4.3±3.8	4.6±4.0	0.607
Body mass index (kg/m^2^)	25.1±3.0	20.6±3.1	<0.001
SBP (mmHg)	137.8±19.0	141.3±16.1	0.158
DBP (mmHg)	74.6±8.0	76.8±7.4	0.037
Smoking	13 (14.0%)	11 (9.9%)	0.369
HCV infection (%)	48 (51.6%)	49 (44.1%)	0.287
Diabetes	50 (53.8%)	55(49.5%)	0.549
PAD	25 (26.9%)	12 (10.8%)	0.003
Albumin (g/dl)	3.8±0.4	3.9±0.4	0.526
Hemoglobulin (mg/dl)	10.3±1.6	10.2±1.6	0.873
GPT (U/L)	18.6±9.4	25.4±41.3	0.093
Fasting blood glucose (mg/dl)	120.8±68.7	119.2±59.9	0.865
Uric acid (mg/dl)	7.5±1.5	7.2±1.3	0.057
Insulin (µIU/ml)	28.1±33.1	18.8±25.3	0.029
C-Peptide	14.0±8.4	10.7±8.5	0.007
HOMA-IR	9.2±12.0	5.2±5.2	0.004
Total Cholesterol (mg/dl)	170.2±41.7	162.8±32.7	0.166
Triglyceride (mg/dl)	179.4±134.3	141.2±83.2	0.018
HDL-cholesterol (mg/dl)	41.9±12.4	47.6±17.3	0.008
LDL-cholesterol (mg/dl)	99.7±28.8	92.4±22.4	0.045
Ln-hsCRP (mg/dL)	1.5±1.3	1.1±1.2	0.021
Ln-Adiponectin (pg/mL)	5.6±0.3	5.7±0.3	0.008
Ln-IL-6	2.9±0.9	2.9±1.1	0.958
Ln-TNF-α(pg/mL)	0.9±1.0	1.0±0.9	0.415
Ln-ADMA (pg/mL)	3.2±0.4	3.3±4.2	0.103
Kt/V	1.81±0.29	1.81±0.34	0.939
ABI	0.96±0.23	1.08±0.16	<0.001
PWV (m/s)	17.9±5.5	18.4±4.4	0.476

There were no significant differences in the levels of serum albumin, hemoglobin, alanine aminotransferase, fasting blood glucose, uric acid, total cholesterol, and ln-transformed IL-6 and TNF-α. However, patients with AO had higher levels of serum insulin, C-peptide, HOMA-IR, low-density lipoprotein cholesterol, triglyceride, and ln-transformed hs-CRP, and lower levels of high-density lipoprotein (HDL) cholesterol and ln-transformed adiponectin (Table 1). Further, those patients with AO had lower levels of ABI (0.96±0.23 vs 1.08±0.16, *P*<0.001). With regard to the role of adequate dialysis, we found no significant difference in the Kt/V values between the 2 patient groups.

Upon analysis of correlations between WC and other variables, WC was found to be significantly positively correlated with the levels of uric acid (*P = *0.002), triglycerides (*P = *0.016), insulin (*P = *0.001), C-peptide (*P = *0.001), HOMA-IR (*P = *0.001), ln-transformed hs-CRP (*P = *0.001), and BMI (*P*<0.001) (Table 2). In addition, WC was significantly negatively correlated with the levels of HDL (*P*<0.001) and ABI (*P = *0.005).

**Table 2:** Pearson correlation coefficients between waist circumference and the other variables in hemodialysis patients (n = 204).

	*r*	*P* Value
Age	0.073	0.296
Body mass index (kg/m^2^)	0.725	<0.001
Blood pressure		
Systolic	−0.019	0.787
Diastolic	−0.055	0.435
Albumin	−0.083	0.236
Glucose	0.016	0.824
Uric acid	0.211	0.002
Plasma lipids		
LDL	0.118	0.092
HDL	−0.298	<0.001
Triglycerides	0.168	0.016
Insulin	0.233	0.001
C-peptide	0.259	0.001
HOMA-IR	0.237	0.001
ABI	−0.198	0.005
PWV (m/s)	−0.005	0.942
Ln-hsCRP (mg/dL)	0.254	<0.001
Ln-TNF-α(pg/mL)	0.010	0.886
Ln-IL-6(pg/mL)	−0.006	0.938
Ln-ADMA (pg/mL)	−0.103	0.179
Ln-Adiponectin (pg/mL)	−0.097	0.166

Multiple logistic regression analysis was performed to evaluate the association of each parameter with AO. After adjusting for age, sex, BMI, and other confounders in model 1, male gender, BMI, and ABI exhibited an independent relationship with AO (*P*<0.05, respectively). Furthermore, male gender, uric acid, HOMA-IR, ln-transformed adiponectin, and ABI were independent factors for AO after excluding the confounder of BMI in model 2 (*P*<0.05, respectively) (Table 3).

**Table 3:** Logistic regression of multiple factors associated with abdominal obesity in hemodialysis patients (n = 204).

Variables	Odds ratio	95% CI	*P* Value
Model 1			
Male (vs Female)	0.273	0.122–0.609	0.002
Body mass index (kg/m^2^)	1.837	1.537–2.195	<0.001
ABI	0.028	0.003–0.263	0.002
Model 2			
Male (vs Female)	0.372	0.195–0.710	0.003
Uric acid	1.401	1.111–1.766	0.004
HOMA-IR	1.056	1.012–1.102	0.012
Ln-Adiponectin (pg/mL)	0.246	0.092–0.657	0.005
ABI	0.028	0.005–0.165	<0.001

Model 1: By using multiple logistic foreward regression analysis, all covariates were used for analysis. Model 2: By using multiple logistic foreward regression analysis, all covariates were used for analysis, except body mass index. CI, confidence interval.

Subsequently, we performed additional logistic regression tests to evaluate the association of each parameter with PAD. Multivariate analysis showed that age, duration of HD, HDL-cholesterol, ln-transformed IL-6, ln-transformed ADMA, and AO were significantly associated with PAD (*P*<0.05, respectively) (Table 4).

**Table 4:** Logistic regression of multiple factors associated with PAD in hemodialysis patients (n = 204).

Variables	Odds ratio	95% CI	*P* Value
Age (yrs)	1.075	1.031–1.120	0.001
HD years	1.212	1.081–1.359	0.001
HDL-cholesterol (mg/dl)	0.938	0.901–0.977	0.002
Ln-IL-6(pg/mL)	1.567	1.033–2.378	0.035
Ln-ADMA (pg/mL)	5.535	1.323–23.155	0.019
AO (vs non-AO)	4.532	1.765–11.639	0.002

AO, abdominal obesity; CI, confidence interval.

## Discussion

There are 2 new major findings of this study. First, AO was found to be correlated with the female gender, higher BMI, and lower ABI levels in our study group. After adjusting the confounding factor of BMI, female gender, lower ABI, higher plasma uric acid, lower ln-transformed adiponectin, and higher HOMA-IR levels independently predicted the presence of AO. This reflects that the nature of AO is insulin resistance. Second, our results show that higher plasma levels of ln-transformed ADMA and ln-transformed IL-6 and lower plasma levels of HDL are associated with PAD, as well as with AO, older age, and longer HD duration. Our findings suggest that increased insulin resistance (HOMA-IR), oxidative stress (ADMA, HDL), and inflammation (IL-6) due to AO are independently associated with PAD in HD patients, which are not analyzed in previous studies [8]–[9].

Intra-abdominal fat and insulin resistance are important causative factors of metabolic syndrome [16], and WC is considered as a simple anthropometric index of intra-abdominal fat accumulation [17]. Although the waist-to-hip ratio is precise for measuring fat distribution [18], WC is a more readily available clinical measure for the estimation of visceral adiposity. Moreover, Burton *et al* suggested that WC may be a simple and reliable clinical tool for the detection of underlying CKD within primary care [19]. In addition, given the complex interaction between adiposity and uremia, a combined screening tool using BMI and WC or WHR is unlikely to provide any additional benefit to risk analysis [20]. In the Second Manifestation of Arterial Disease (SMART) study, Gorter *et al* [21] found that metabolic syndrome was present in 58% of PAD patients, with women showing a higher prevalence than men (65% vs 55%). Metabolic syndrome increases the risk of cardiovascular mortality [22] and worsening PAD [23]. Our data are in agreement with those of previous studies that have defined AO as having a WC of 80 cm or more in women and a WC of 90 cm or more in men according to the Asia Pacific World Health Organization guidelines. It was observed that women with AO were more insulin resistant and dyslipidemic. Moreover, women had lower plasma ln-transformed adiponectin levels and lower ABI values than men. To the best of our knowledge, this is the first study demonstrating a correlation between AO and decreased ABI in HD patients.

By multivariate age-adjusted logistic regression, our data showed that AO, and not BMI, is associated with a 4-fold risk of developing PAD (OR 4.532, 95% CI, 1.765–11.639, *P* = 0.002). Visceral fat is the most metabolically active fat store and a key factor in the development of insulin resistance, type-2 diabetes, and atherosclerosis [24]. It is also associated with inflammation and oxidative stress [25]. Central obesity, but not BMI, has previously been associated with PAD in a cohort of elderly men [8]. Similarly, in a study of elderly participants from the Osteoporotic Fractures in Men study, waist-to-hip ratio, but not BMI, was associated with low ABI. In the German cohort of the Reduction of Atherothrombosis for Continued Health registry, 50% of patients with PAD had AO [9]. Obesity has previously been associated with the severity of PAD [26]. Obese patients report more calf pain than the general population, and obese patients who undergo surgical treatment for obesity have a lower risk of developing calf pain [27]. Taken together, the literature suggests that body composition, particularly for persons with increased central fat, may indicate increased risk for PAD. Therefore, obese patients or those whose clearance of cytokines is impaired, as in advanced CKD or HD patients, may be prone to insulin resistance and accelerated atherosclerosis. In addition to general poputation, the present study extends these findings by identifying an association between AO and PAD in HD patients.

This study provides some evidence for the possible pathophysiological mechanisms underlying the relationship between AO (high WC), insulin resistance, and PAD. IL-6 is one of the most studied cytokines associated with PAD and is shown to contribute to a wide-spectrum of physiological and pathophysiological processes [28]. IL-6 enhances the production of CRP and TNF-α in the liver, in addition to up-regulating cellular adhesion molecule expression by the endothelial and smooth muscle cells, which are considered relevant to atherosclerotic progression [29]. IL-6 also has been shown to increase leukocyte recruitment into atherosclerotic arterial cell walls by stimulating endothelial cell chemokine release and up-regulating intercellular adhesion molecule-1 on smooth muscle cells. In addition, IL-6 stimulates smooth muscle cells to develop into foam cells [30]. Clinically, high levels of IL-6 (and its hepatic bio-product, CRP) are associated with increased risks of coronary and peripheral atherosclerosis [31]. The Edinburgh artery [32] and InCHIANTI [33] studies have completely assessed the role of IL-6 as a predictor of PAD. Furthermore, IL-6 has been found to be associated with PAD severity [34], and a previous study demonstrated that polymorphisms in the IL-6 gene were associated with increased PAD susceptibility in type 2 diabetics [35].

Interestingly, we identified for the first time to found statistically elevated levels of the proinflammatory cytokine, IL-6, and oxidative stress markers, ADMA, in patients with PAD compared to that in non-PAD controls, demonstrating that there is a characteristic pattern of phlogistic biomarkers in subjects with PAD. We hypothesize that these analytic measures could be useful to predict the morbidity for PAD. We postulate that some of these analytes could be considered as indicators and/or predictors of morbidity for PAD considering that inflammatory cytokines are surely involved both in the mediation and progression of endothelial dysfunction on the arterial wall of the peripheral arteries. Finally, we believe that inflammatory biomarker levels should be considered as a target of different medical or interventional approaches used to treat patients with PAD. It is known that physical training was effective in lowering high plasma levels of such inflammatory bio-markers [36]. Moreover, it was effective against inflammation; this represents a crucial goal for medicated stents that are still routinely applied for coronary arteries and that have been recently postulated as useful interventional method for the PAD [37]. Therefore, demonstrating the key role of these cytokines could aid in the diagnosis of PAD, and they can be used as a means of developing novel treatment modalities for the prevention and management of PAD by antagonizing the effects of these inflammatory mediators and/or oxidative stress markers.

Increased ADMA may affect vascular function and structure through various mechanisms. A previous study has shown that elevation in ADMA may at least in part cause endothelial nitric oxide synthase (eNOS) uncoupling, increase vascular superoxide levels, and contribute to oxidative stress [38], which per se may be a major mechanism of vascular impairment [39]–[40]. Increased levels of ADMA also reduce bioavailability of nitric oxide (NO) and cause endothelial cell dysfunction [38] by blocking all 3 isoforms of NOS and enhancing NO degradation due to eNOS-mediated superoxide production. It has been demonstrated that ADMA causes vascular arteriosclerotic lesions in an eNOS-independent manner. Direct upregulation of the angiotensin-converting enzyme and increased oxidative stress via the angiotensin II type 1 receptor might also be involved in the long-term vascular effects of ADMA [41]. However, elevated ADMA levels promote endothelial-monocyte interaction [42], related to carotid intima-media thickness [43], and correlate with severity of PAD [44], suggesting that an increase in ADMA levels is associated with critical processes in atherogenesis.

We found a statistically significant correlation between age and PAD. This may be caused by the fact that the oldest subjects were aged 70 years and the prevalence of PAD almost doubles after the age of 70 [45]. An association between PAD and duration of dialysis was found in a previous study dealing with PAD risk factors in HD patients [5]. Our study also showed the association of HDL with PAD in multivariate analysis, and this was different from the general population in previous studies [8]. High hs-CRP as well as low HDL cholesterol was associated with low ABI in 2 cross-sectional studies [46]–[47]. Recent studies focused on the loss of antioxidant and anti-inflammatory effects of HDL in dialysis patients [48]–[49]. In accordance with our report, a relationship that might associate either the HD process or the uremic milieu with increased atherosclerotic disease burden has been suggested.

There are limitations that need to be acknowledged. First, the cross-sectional nature of our study does not allow inferences on causality; therefore, implications on possible mechanisms should be regarded as hypotheses. Second, this study was monocentric, and it demonstrates limitations such as the small number of the enrolled subjects. However, we would like to emphasize that these subjects were diagnosed from a large HD population screened for detecting PAD; further, they were asymptomatic and the presence of an initial disorder of the arteries in the lower limbs was unknown.

To our knowledge, this is the first study providing evidence that AO is positively associated with PAD in the Taiwanese HD population. Serum adiponectin levels and ABI are significantly associated with AO, especially in women. AO, elevated serum ln-transformed ADMA levels, and ln-transformed IL-6 levels were independent predictors of the presence of PAD in HD patients.
